# Serological Evidence of SARS-CoV-2 Exposure in Domestic Dogs and Cats, Thailand: Detection of SARS-CoV-2 Omicron Variant in Dogs Living in COVID-19-Positive Households

**DOI:** 10.1155/2024/9938523

**Published:** 2024-02-20

**Authors:** Supassama Chaiyawong, Kamonpan Charoenkul, Waleemas Jairak, Kitikhun Udom, Ekkapat Chamsai, Navapon Techakriengkrai, Kamol Suwannakarn, Alongkorn Amonsin

**Affiliations:** ^1^Department of Veterinary Public Health, Faculty of Veterinary Science, Chulalongkorn University, Bangkok 10330, Thailand; ^2^Emerging and Re-Emerging Infectious Diseases in Animals, Center of Excellence, Faculty of Veterinary Science, Chulalongkorn University, Bangkok 10330, Thailand; ^3^Department of Veterinary Microbiology, Faculty of Veterinary Science, Chulalongkorn University, Bangkok, Thailand; ^4^Department of Microbiology, Faculty of Medicine Siriraj Hospital, Mahidol University, Bangkok, Thailand

## Abstract

SARS-CoV-2 causes the coronavirus disease 2019 (COVID-19) pandemic. Cross-species transmission of SARS-CoV-2 from humans to domestic animals has been reported. In this study, we conducted a serological survey and molecular investigation of SARS-CoV-2 infection in domestic dogs and cats in Bangkok and the vicinities from January 2021 to August 2022. A total of 2,664 serum samples were examined for antibodies against SARS-CoV-2 using nucleocapsid protein-based ELISA (NP-ELISA). Our result showed 2.28% (33/1,446) seropositivity in dogs and 1.81% (22/1,218) in cats. The positive NP-ELISA serum samples were confirmed using a surrogate virus neutralization test (sVNT). Of 55 seropositive samples by NP-ELISA, two dogs and 19 cats were confirmed seropositive by sVNT. Our result supported the serological evidence of SARS-CoV-2 exposure in domestic dogs and cats. We also investigated SARS-CoV-2 infection by real-time RT–PCR in 156 domestic dogs and cats in COVID-19-positive households. Our result showed active SARS-CoV-2 infection in a dog living with COVID-19 positive owner. Genetic and phylogenetic analysis of the SARS-CoV-2 from the dog and its owner confirmed the SARS-CoV-2 variant Omicron BA.2. It is the first report of SARS-CoV-2 Omicron variant in pet living in COVID-19-positive household in Thailand.

## 1. Introduction

Coronavirus disease 2019 (COVID-19), caused by severe acute respiratory syndrome coronavirus 2 (SARS-CoV-2), is a pandemic disease. COVID-19 has led to more than 768 million confirmed human cases, with 6.95 million deaths worldwide (WHO, 2023). The accumulated mutations in the SARS-CoV-2, especially in the spike protein, led to viral evolution and subsequently generated new variants. Previously, the World Health Organization (WHO) has classified SARS-CoV-2 into five variants of concern (VOCs), eight variants of interest (VOI), and 15 variants under monitoring (VUM) (WHO 2022). The VOC variants identified were Alpha (B.1.1.7), Beta (B.1.351), Gamma (P.1), Delta (B.1.617.2), and Omicron (B.1.1.529). Since November 2021, the Omicron variant has gained public health concerns as the dominant variant infected globally and evolved into several sublineages (BA.1, BA.2, BA.3, BA.4, BA.5). In March 2023, the WHO updated variant tracking system by classification of Omicron sublineages. There are currently no SARS-CoV-2 variants to be classified as VOCs. However, descendent variants of Omicron sublineages are classified as VOIs (BQ.1, BA.2.75, XBB, XBB.1.5) and VUMs (BF.7, BA.2.3.20, CH.1.1, BN.1, XBC, XAY). In Thailand, at least five SARS-CoV-2 epidemic waves have been reported. The SARS-CoV-2 variant A.6 caused the predominant outbreak in the 1^st^ wave (March–April 2020), B.1.36.16 in the 2^nd^ wave (December 2020–January 2021), B.1.1.7 (Alpha) in the 3^rd^ wave (April–June 2021), B.1.617.2 (Delta) in the 4^th^ wave (July–December 2021), and B.1.1.529 (Omicron) in the 5^th^ wave (from January 2022 onward) [1].

Evidence of SARS-CoV-2 spillover from humans to animals has been reported in several animal species, including domestic dogs and cats. The World Organization for Animal Health (WOAH) reports SARS-CoV-2 infection in dogs and cats in at least 36 countries, including Thailand. Moreover, SARS-CoV-2 Omicron variant infection in domestic dogs and cats was reported in nine countries. Several studies reported serological surveys of SARS-CoV-2 infection in dogs and cats. For example, there are reports of seropositivity for SARS-CoV-2 in dogs and cats from China [2, 3], France [4, 5], Italy [6–8], Spain [9], Germany [10, 11], Turkey [12], Argentina [13], Serbia [14], Poland [15], and the USA [16]. In Thailand, during the 1^st^ and 2^nd^ waves of COVID-19 outbreaks, a serological study showed low seropositivity in dogs (1.66%) and cats (0.36%) [17]. During the 3^rd^ and 4^th^ waves of COVID-19 outbreaks, SARS-CoV-2 infection caused by Alpha (B.1.1.7) and Delta (B.1.617.2) variants have been reported in dogs and cats [18, 19].

In this study, we conducted a serological survey for SARS-CoV2 antibodies in domestic dogs and cats in Bangkok and the vicinities from January 2021–August 2022, corresponding to 2^nd^ to 5^th^ wave of COVID-19 outbreaks in Thailand. We also investigated SARS-CoV-2 infection by real-time RT–PCR in domestic dogs and cats in COVID-19-positive households. The study provided serological evidence of SARS-CoV-2 exposure in domestic dogs and cats and reported the first description of a SARS-CoV-2 Omicron variant in pets living in COVID-19-positive households in Thailand.

## 2. Materials and Methods

### 2.1. Serological Survey of SARS-CoV-2 in Domestic Dogs and Cats

Blood samples were collected from domestic dogs and cats during routine visits to the Chulalongkorn University Small Animal Hospital from January 2021 to August 2022 (a period of 20 months). The Chulalongkorn University Animal Care and Use protocol (CU-IACUC No. 2031035) approved and authorized the sample collection. Total of 2,664 blood samples from dogs (*n* = 1,446) and cats (*n* = 1,218) were included for serological testing. The serum samples were collected and stored at −20°C until serological testing.

### 2.2. Detection of SARS-CoV-2 Antibodies Using the Nucleocapsid Protein-Based ELISA (NP-ELISA)

This study used the commercially available ELISA ID Screen® SARS-CoV-2 Double Antigen Multi-species ELISA kit (ID VET, Montpellier, France) to detect SARS-CoV-2 antibodies. The ELISA assay was designed to identify IgG antibodies against the nucleocapsid protein (NP) of the SARS-CoV-2 virus in sera of dogs and cats. The NP-ELISA test was conducted following the manufacturer's instructions. To interpret the result, a serum with an S/P% of >60% was defined as seropositive, an S/P% of 50%–60% was considered suspect, and an S/P% of <50% was considered seronegative.

### 2.3. Detection of SARS-CoV-2 Antibodies Using the SARS-CoV-2 Surrogate Virus Neutralization Test (sVNT)

To detect the presence of neutralizing SARS-CoV-2 antibody, 63 positive and suspected serum samples (consisting of 38 samples from dogs and 25 from cats) were tested using the SARS-CoV-2 Surrogate Virus Neutralization Test (sVNT) Kit (cPass™ Technology, GenScript Biotech, China). This sVNT assay detects the binding of the purified receptor binding domain (RBD) of SARS-CoV-2 viral spike protein and host ACE2 receptor. To interpret the result, the sample with % inhibition ≥20% is positive for the presence of SARS-CoV-2 neutralizing antibody.

## 3. Investigation of SARS-CoV-2 Infection in Domestic Dogs and Cats from COVID-19-Positive Households

From December 2021 to August 2022, we investigated SARS-CoV-2 infection in domestic dogs and cats from COVID-19-positive households in Bangkok and the vicinities. In total, 156 animals were collected from 49 COVID-19-positive households (dogs *n* = 96, cats *n* = 60). All 156 animals from COVID-19-positive households have been in contact with SARS-CoV-2 infected owners. In addition, 316 samples were collected from unknown-status households (dogs; *n* = 221, and cats; *n* = 95). The sample collection was conducted based on the convenience and willingness for COVID-19 testing of pet owners and animal hospital staff. The samples (nasal, oral, and rectal swabs) were collected.

All samples were tested for the presence of SARS-CoV-2 RNA by real-time RT–PCR. When the SARS-CoV-2 positive sample was identified, an epidemiological investigation of the corresponding household was carried out. After SARS-CoV-2 RNA detection, an additional 16 swab samples (nasal, oral, rectal, and hair swabs) were collected on Days 1, 3, 5, and 7 from positive animals. Serum samples (*n* = 5) of the positive animals were collected on Days 3, 7, 14, 22, and 31 for serological tests. Moreover, epidemiological information and nasal swab sample (*n* = 1) of the owner were collected to confirm the SARS-CoV-2 infection.

### 3.1. Detection of SARS-CoV-2 RNA Using Real-Time RT–PCR

RNA extraction from swab samples was performed using GENTi, Automated Nucleic Acid Extraction System (GeneAll®, Seoul, Korea). To detect SARS-CoV-2, a real-time RT–PCR assay, with primers and probes specific to the E and RdRp genes, was used following WHO recommendations [20]. The real-time RT–PCR was carried out using the SuperScript® III Platinum® One-Step Quantitative RT-PCR System (Invitrogen®). Synthesized oligonucleotides carrying the target sites (E and RdRp genes) were used as the positive control. According to the WOAH definition, the confirmation of animal cases of SARS-CoV-2 infection requires at least two specific targets to test positive (https://www.woah.org/app/uploads/2022/08/en-sars-cov-2-surveillance.pdf) (OIE, 2021).

### 3.2. Genetic Characterization, Mutation Analysis, and Phylogenetic Analysis of SARS-CoV-2 by Whole-Genome Sequencing

In this study, nasal swab samples from a dog (CU28424) and the pet owner (CUh10001) that tested positive for SARS-CoV-2 RNA were subjected to whole genome sequencing using Oxford Nanopore with ARTICS nCoV-2019 sequencing protocol V3 (LoCost) (Oxford nanopore technologies, Oxford, UK). The parameters (minimum read length ≥500 nt and read quality ≥7) were used to filter and validate nucleotide sequences. The Fastq format sequences were assembled by matching to reference Omicron SARS-CoV-2 sequence using the Qiagen CLC Genomics Workbench version 20.0.4 software (QIAGEN, CA, USA).

The whole-genome sequences of SARS-CoV-2 from the dog and its owner were subjected to lineage identification by using the Phylogenetic Assignment of Named Global Outbreak Lineages (PANGOLIN) (https://cov-lineages.org/resources/pangolin.html). A phylogenetic analysis of Omicron SARS-CoV-2 was performed. The maximum likelihood tree was generated using IQ-TREE 2 [21] with default heuristic search options and 1,000 bootstrapping replicates, using the TIM + I + *Γ* model of nucleotide substitution [22]. The tree was visualized by iTOL [23]. For genetic mutation analysis, the deduced amino acids of each gene of the SARS-CoV-2 were analyzed using the MEGA 7 program (https://www.who.int/activities/tracking-SARS-CoV-2-variants).

### 3.3. Detection of Antibodies for SARS-CoV-2 in Animals

To detect SARS-CoV-2 antibodies, the SARS-CoV-2 Surrogate Virus Neutralization Test (sVNT) Kit was used to detect specific RBD neutralizing antibodies against the SARS-CoV-2. An additional assay, pseudotype virus neutralization test (pVNT), was used to detect non-RBD neutralization antibodies [18].

## 4. Results

### 4.1. Serological Evidence of SARS-CoV-2 Infection in Domestic Dogs and Cats

In this study, a total of 2,664 serum samples from dogs (*n* = 1,446) and cats (*n* = 1,218) were collected from January 2021 to August 2022. The sample collection period begins from 2^nd^ to the 5^th^ wave of COVID-19 outbreaks in Thailand. The number of animal samples and the results of SARS-CoV2-2 antibody detection in dogs and cats each month are shown in Table 1. The NP-ELISA for detecting antibodies against the N-protein of SARS-CoV-2 revealed that 2.06% (55 out of 2,664) of animals tested positive (S/P% >60%), and 0.3% (8 out of 2,664) of animals were suspected (S/P% = 50%–60%). In detail, 2.28% (33 out of 1,446) of dogs and 1.81% (22 out of 1,218) of cats were seropositive, while 0.35% (5 out of 1,446) of dogs and 0.25% (3 out of 1,218) of cats were suspected. It is noted that SARS-CoV-2 antibodies could be detected year-round during the survey period, except in April 2021 and November 2021. The highest seropositivity for SARS-CoV-2 antibodies was found in May 2022, with 8.51% (8 out of 94) by NP-ELISA and 4.26% (4 out of 94) by sVNT (Table 1).

For serum samples from dogs, the highest SARS-CoV-2 seropositivity by NP-ELISA was 7.27% (4 out of 55) in March 2022. The positive-canine sera by NP-ELISA (*n* = 38) were also tested for neutralizing antibodies by sVNT. SARS-CoV-2 neutralizing antibodies could be detected in two dogs (2/38; 5.26%) in March 2022 (*n* = 1) and April 2022 (*n* = 1) with 30.36% and 42.18% inhibition, respectively. For serum samples from cats, the highest SARS-CoV-2 seropositivity was observed in May 2022 by NP-ELISA at 11.11% (5 out of 45) and by sVNT at 8.89% (4 out of 45). The NP-ELISA positive and suspected samples of cats (*n* = 25) were subjected to a test for neutralizing antibodies by sVNT. Interestingly, our result showed that samples of 19 cats (19/25; 76%) were seropositive (Table 1).

To monitor the persistence of SARS-CoV-2 antibodies, three SARS-CoV-2 seropositive animals were followed up. During the 4 to 8-month follow-up, three cats were positive for SARS-CoV-2 antibodies by NP-ELISA and sVNT. For example, cat 1 (CU12324) was seropositive for at least 8 months with high seropositivity (NP-ELISA S/P% 270%–304%; sVNT % inhibition 85%–97%). Similarly, cat 2 (CU13867) and cat 3 (CU14208) showed seroconversion (NP-ELISA S/P% 520%–670%; sVNT % inhibition 96%–97%) for approximately 4 months (Table 2).

## 5. SARS-CoV-2 Infection in COVID-19-Positive Households

**Figure 1:**
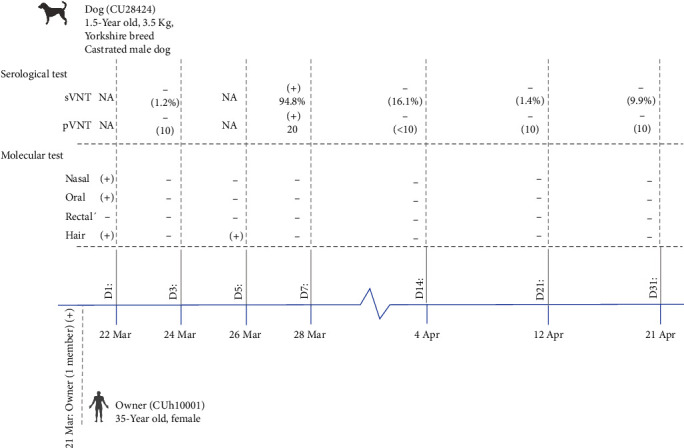
Timeline of SARS-CoV-2 detection in domestic dog and owner in this study.

From December 2021 to August 2022, we investigated SARS-CoV-2 infection in domestic dogs and cats from COVID-19-positive households (Table S1). In total, 156 animals from 49 COVID-19-positive households (dogs *n* = 96, cats *n* = 60) were tested for SARS-CoV-2 RNA. On 22 Mar 2022, we detected SARS-CoV-2 RNA in a dog from a household of SARS-CoV-2 infected patients. The SARS-CoV-2 positive dog (CU28424), 1.5-year-old Yorkshire Terrier, a neutered male dog, did not show any clinical signs. Swab samples, including nasal, oral, rectal, and hair swabs, were collected from the dog on Days 1, 3, 5, and 7 after the owner was confirmed positive for COVID-19. The nasal and oral swab samples from the dog (CU28424) tested positive on Day 1 (E gene: Ct 32.37 and RdRp gene: Ct 34.33), and the hair swab samples tested positive on Days 1 and 5 (E gene: Ct 30.18 and RdRp gene: Ct 31.82; Table 3). On 21 March 2022, the owner (CUh10001), 35-year-old-female, tested positive for SARS-CoV-2 by antigen test kit and confirmed by RT–PCR at a local hospital. On 24 March 2022, we collected a nasal swab sample from the owner, and the sample tested positive (E gene: Ct 25.63 and RdRp gene: Ct 25.03; Figure 1 and Table 3).

**Figure 2:**
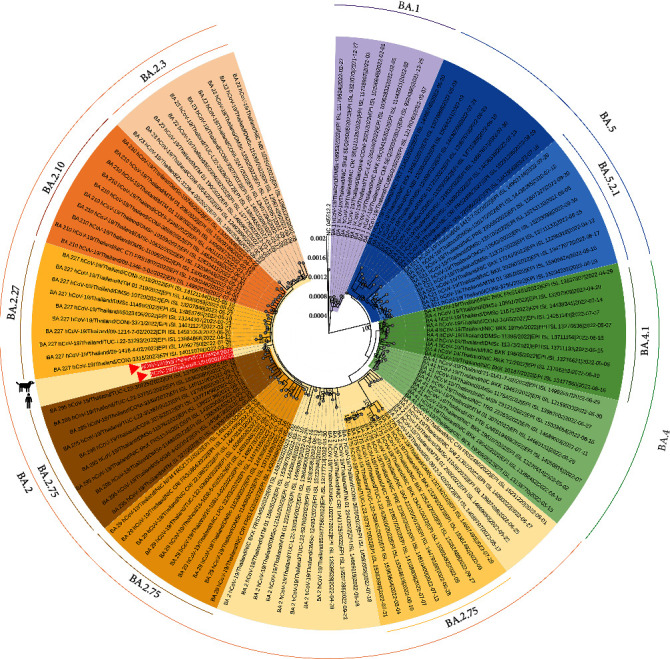
The maximum likelihood tree of Omicron variant SARS-CoV-2 from the dog, its owner, and human in Thailand. Color represents sublineage of Omicron, including purple (BA.1), light–dark yellow (BA.2), light–dark green (BA.4), and light–dark blue (BA.5). Red arrows represent SARS-CoV-2 of the dog and its owner in the study.

In this study, whole-genome sequences of nasal samples obtained from the dog (CU28424) and the owner (CUh10001) were available and submitted to the database under the GenBank accession numbers: OP862443 and OP862444, and GISAID numbers: EPI_ISL_15833374 and EPI_ISL_15833476. The whole genome sequences consist of 92.7% and 98.9% length coverage of the whole genome, respectively. The identification of the SARS-CoV-2 variant was performed using the program PANGOLIN, and the whole genome sequences of the dog and its owner were assigned as the SARS-CoV-2 Omicron BA.2 variant. The phylogenetic analysis also showed that the dog and its owner SARS-CoV-2 clustered to an Omicron BA.2 (B.1.1.529 + BA.2) variant sequence. It is noted that the Omicron variant has been reported in Thailand since November 2021 and, at present, extended to the predominant variant. Among BA.2, several sub-lineages, BA.2.10, BA.2.27, BA.2.3, and BA.2.75, have been reported in the country (Figure 2).

Genetic analysis showed that the dog SARS-CoV-2 nucleotide sequence (CU28424) has 99.99% similarity of nucleotide sequence (27,292 bp) compared to those of the owner SARS-CoV-2 (CUh10001). The analysis of the spike gene and internal genes of the SARS-CoV-2 sequences retrieved from the dog and its owner showed that the mutations were identical to the Omicron BA.2 variant, except position 440 at spike protein contained no mutation (which was the same as the Wuhan variant). Additionally, position 366 at NP protein had one amino-acid deletion. Compared to the reference Omicron BA.2 from canine (EPI_ISL_13101428), the dog SARS-CoV-2 nucleotide sequence has 99.96% similarity (26,707 bp). Only nine nucleotide substitutions led to four amino-acid mutations were observed in ORF1ab (A1938V, V2909A), ORF3a (F140L), and N gene (E19G) (Table 4 and Table S2).

The serological test showed that the dog (CU28424) was positive for SARS-CoV-2 antibodies by sVNT (94.8% inhibition) and pVNT (titer = 1 : 20) at Day 7. Both serological tests confirmed the seroconversion of the SARS-CoV-2-positive animals (Table 5).

## 6. Discussion

This study conducted a serological survey for SARS-CoV-2 antibodies from January 2021 to August 2022, corresponding to Thailand's 2^nd^ to 5^th^ wave of COVID-19 outbreaks. Our results showed that 2.28% of dogs and 1.81% of cats were seropositive for NP-ELISA, and 0.14% of dogs and 1.56% of cats were seropositive for sVNT. The positivity of SARS-CoV-2 exposure reported here was higher than previous serological studies conducted from April 2020 to December 2020, corresponding to the 1^st^ and 2^nd^ waves of COVID-19 outbreaks in Thailand [17]. The previous serological study reported 1.66% seropositivity in dogs and 0.36% seropositivity in cats by NP-ELISA, but none could be confirmed by sVNT. Our findings suggested that increasing COVID-19 infection in humans could lead to a higher chance of COVID-19 exposure in the pets. Due to dogs and cats being in close contact with humans, spillover of the SARS-CoV-2 virus from infected patients frequently occurs. In this study, the discrepancy between NP-ELISA and sVNT results was observed; this may be due to the low sensitivity of NP-ELISA. It should be noted that the performance of NP-ELISA and sVNT has been documented. The sensitivity of the NP-ELISA was low (23%–36% in dogs, 63% in cats), but the specificity was high (85%–99% in dogs, 96% in cats) [24]. On the other hand, the sensitivity and specificity of sVNT were high (98.8%–100% sensitivity; 98.8%–100% specificity) [25].

The prevalence of seropositivity for SARS-CoV-2 in dogs was 0.14% which was lower than that of the previous studies, including 0.78% in Wuhan, China [3], 1.5% in Italy [8], 3.79% in France [4], and 1.06% in Germany and Italy [7, 10]. On the other hand, in other studies, SARS-CoV-2 antibodies in dogs could not be detected using neutralizing assays [5, 6, 16]. The seropositivity of cats was 1.56%, which was lower than that of the previous studies, including 9.19% in Wuhan, China [2], 2.60% in Italy [8], 6.25% and 9.30% in France [4, 5], 6.27% in the USA [16], and 1.9% in Germany [10]. In this study, the prevalence of seropositivity was higher than that in some previous studies in Germany (0.22%) [11] and Spain (1.17%) [9]. Notably, variations of seropositivity of SARS-CoV-2 in domestic dogs and cats depend on many factors, including exposure to COVID-19-positive patients, time of sample collection, and antibody detection technique.

Our findings showed the concordance between the increasing COVID-19-confirmed human cases and seropositivity in domestic dogs and cats. In Thailand COVID-19 human cases were approximately 23,000 cases in the 2^nd^ wave, 235,000 cases in the 3^rd^ wave, 1.9-million cases in the 4^th^ wave, and 2.4-million cases in the 5^th^ wave (as of August 2022). SARS-CoV-2 seropositivity in dogs and cats in Thailand in the 2^nd^ wave was 0% [17, 26]. Comparable to this study, seropositivity was 0% in the 3^rd^ wave (April–June 2021), 0.86% in the 4^th^ wave (July–December 2021), and 1.74% in the 5^th^ wave (January–August 2022).

In this study, we monitored the persistence of SARS-CoV-2 antibodies in three SARS-CoV-2 seropositive animals. Our result indicated that the cats could present SARS-CoV-2 antibodies lasting up to 8 months. It is noteworthy that reinfection of these cats cannot be ruled out. Our findings supported the previous studies that naturally infected animals have antibody responses to SARS-CoV-2 for more than 8 months (in dogs) and more than 16 months (in cats) [27, 28].

In Thailand, SARS-CoV-2 infections in dogs and cats have been reported, and the variants of SARS-CoV-2 causing COVID-19 in animals correspond to the predominant variants during the COVID-19 outbreaks in humans. For example, during the 3^rd^ wave of the COVID-19 outbreak, the SARS-CoV-2 Alpha variant was detected in three dogs and seven cats [19]. During the 4^th^ wave of the COVID-19 outbreak, the Delta variant was detected in a dog and two cats [18, 19]. In this study, during the 5^th^ wave of the COVID-19 outbreak, the Omicron variant was detected in a dog. The nasal and oral swab samples from the dog (CU28424) tested positive on Day 1, and the hair swab samples tested positive on Days 1 and 5, suggesting environmental contamination of the viruses from infected animals could not be ignored. The infected dog represented 0.64% (1/156) of the dogs and cats living with COVID-19-positive owners and 2% (1/49) of COVID-19 households. Notably, the occurrence of SARS-CoV-2 Omicron variant infection in domestic pets in this study was lower than that of the Alpha and Delta variant infection in domestic dogs previously reported [18, 19]. This might be due to the low virulence of the variant and the short period of viral shedding [29–33]. It is noted that the COVID-19-positive dog (CU28424) did not show any clinical signs similar to the previous report of Omicron variant infection in dogs and cats in Spain [34]. In contrast, domestic pets infected with other variants showed no clinical signs [29, 32], mild clinical signs (lethargic, sneezing, dry cough, watery diarrhea) [30, 31, 35], and severe clinical signs [36]. In a previous experimental study, the cats inoculated with the Omicron showed subclinical signs and shed the virus lower than those inoculated with B.1 (D614G) and Delta variants. The study concluded that the Omicron infection in domestic pets was less pathogenic than the other VOCs [35]. Another experimental study on Omicron infection in a hamster model showed that the Omicron was less pathogenic in the trachea, bronchi, and lungs [37]. While, the experimental Omicron-infected mink showed mild to moderate clinical signs, e.g., lethargy, anorexia, diarrhea, nasal and lacrimal discharge, and sneezing. Moreover, mink-to-mink transmission has been observed [38].

In this study, we conducted serological tests by sVNT and pVNT assays. Both sVNT and pVNT were used to confirm SARS-CoV-2 positive and suspected serum samples. sVNT detects specific receptor binding domain (RBD) neutralizing antibodies, whereas pVNT detects non-RBD neutralizing antibodies. The advantage of using both assays was that the pVNT test could be used to confirm doubtful results by the sVNT test. For example, in our previous study the sample with a low %inhibition value (18.82%) by sVNT (cutoff value >20%) was tested positive for pVNT [18]. Our result showed that the Omicron-infected dog possessed antibodies against SARS-CoV-2 on Day 7. However, the antibody titer was low in sVNT and pVNT and could not be detected on Days 14, 21, and 31. Comparable to the previous study of the Omicron infection in dogs and cats did not show neutralizing antibodies (VNT) or nonneutralizing antibodies (ELISA) [34]. Unlike previous studies of the Alpha and Delta variant infection in dogs and cats, SARS-CoV-2 antibodies developed 7–14 days after exposure and prolonged for 2–3 months [8, 32, 39]. The results of low antibody titer were in concordance with an experimental study in cats in which the Omicron-induced neutralizing antibodies were lower and delayed than the other SARS-CoV-2 variants [35]. The explanation might be that the mutations at the spike protein of the Omicron could help its high-binding affinity with ACE2 but could reduce the neutralization ability of the monoclonal antibodies [40–42].

Phylogenetic analysis of the whole genome of SARS-CoV-2 showed that the Omicron from the dog (CU28424) and its owner (CUh10001) belonged to Omicron subvariant BA.2. Notably, the Omicron subvariant BA.2 was the predominant lineage in Thailand during the time of virus detection in the dog. Mutation analysis of the whole genome showed no variabilities of the amino acids between the Omicron from dog and owner, and the viruses had amino acid mutations similar to those of the Omicron BA.2 lineage (in at least 29 positions at spike protein and 51 positions at whole genome) (https://www.who.int). Nevertheless, the Omicron from the dog and its owner in this study contained asparagine (N440) in spike protein, while the Omicron in the previous study contained lysine (K440). Similarly, N440 was observed in some Omicron variants from humans (EPI_ISL_13338973, EPI_ISL_12678854, EPI_ISL_11889791). This N440K mutation is in the receptor-binding domain and could result in viral escape from a subset of neutralizing antibodies [40]. Another mismatch mutation was observed in the NP protein in which threonine (T) deletion at position 366 was found in the Omicron in this study but not in the other reference viruses. However, the function of 366del is unknown and needs further investigation.

In conclusion, this study showed evidence of SARS-CoV-2 antibodies in domestic dogs and cats. Our result supports the evidence that SARS-CoV-2 exposure occurred in Thailand's domestic dog and cat populations. This study is also the first to reveal the SARS-CoV-2 Omicron variant infection in a domestic dog in Thailand. The genome sequences of the viruses from a dog and its owner are almost identical and clustered with the Omicron subvariant BA.2. The Omicron-infected dog developed no clinical signs and shed the virus in a short period. The animal also developed low SARS-CoV-2 antibody titer. Our result highlighted the importance of active molecular and serological surveillance of SARS-CoV-2 in domestic animals, especially in close contact with COVID-19 owners. This information will help to establish the guidelines and recommendations for pet owners and vet practitioners on COVID-19 prevention and control.

## Figures and Tables

**Table 1:** Detail of serum collected from dogs and cats and serological test for SARS-CoV-2 antibodies by ELISA and sVNT.

Month	Sample	Dog				Cat			
		ELISA positive (suspected)/test	(%)	sVNT	(%)	ELISA positive (suspected)/test	(%)	sVNT	(%)
Jan-21	190	2(1)/103	1.94	0/3	0	1/87	1.15	0/1	0
Feb-21	165	3(1)/101	2.97	0/4	0	0/64	0	0	0
Mar-21	181	3/101	2.97	0/3	0	0/80	0	0	0
Apr-21	140	0/89	0	0/0	0	0/51	0	0	0
May-21	171	3/92	3.26	0/3	0	0/79	0	0	0
Jun-21	198	3/108	2.78	0/3	0	0/90	0	0	0
Jul-21	224	1/130	0.77	0/1	0	0/94	0	0	0
Aug-21	192	3(1)/116	2.59	0/4	0	1/76	1.32	1/1	1.32
Sep-21	96	1/49	2.04	0/1	0	2(2)/47	4.26	3/4	6.38
Oct-21	106	0(1)/55	0	0/1	0	2/51	3.92	1/2	1.96
Nov-21	100	0(1)/50	0	0/1	0	0/50	0	0/0	0
Dec-21	97	0/50	0	0/0	0	2/47	4.26	2/2	4.26
Jan-22	98	1/46	2.17	0/1	0	1(1)/52	1.92	1/2	1.92
Feb-22	102	1/48	2.08	0/1	0	2/54	3.70	2/2	3.70
Mar-22	101	4/55	7.27	1/4	1.82	1/46	2.17	1/1	2.17
Apr-22	104	3/54	5.56	1/3	1.85	1/50	2.00	1/1	2.00
May-22	94	3/49	6.12	0/3	0	5/45	11.11	4/5	8.89
Jun-22	96	1/47	2.13	0/1	0	0/49	0	0/0	0
Jul-22	104	1/51	1.96	0/1	0	1/53	1.89	1/1	1.89
Aug-22	105	0/52	0	0/0	0	3/53	5.66	2/3	3.77
Total	2,664	33(5)/1,446	2.28	2/38	0.14	22(3)/1,218	1.81	19/25	1.56

**Table 2:** Detail of serological test results of positive animals in serological survey at different time points.

Positive cases	Blood collection date	ELISA (S/P%)	sVNT (inhibition %)
Cat 1 (CU12324)	28 Aug 2021	270.73	97.4
	15 May 2022	304.06	85.78

Cat 2 (CU13867)	16 Dec 2021	520.76	96.59
	28 April 2022	637.63	96.78

Cat 3 (CU14208)	2 Feb 2022	670.00	96.52
	31 Mar 2022	666.12	96.44
	26 May 2022	669.23	97.28

**Table 3:** Result of SARS-CoV-2 detection from swab samples of dog and owner by real-time RT–PCR.

ID	Species	Age	Sample	Real-time RT–PCR result (Ct)							
				Day 1^a^		Day 3		Day 5		Day 7	
				E^b^	RdRp^b^	E	RdRp	E	RdRp	E	RdRp
CU28424	Dog	1.5 Y	Nasal	32.37^*∗*^	33.01	—	—	—	—	—	—
			Oral	—	34.33	—	—	—	—	—	—
			Rectal	—	—	—	—	—	—	—	—
			Hair	30.18	31.82	—	—	33.68	30.95	—	—

CUh10001	Owner	35 Y	Nasal	N/A	N/A	25.63^*∗*^	25.03	N/A	N/A	N/A	N/A
			Oral	N/A	N/A	34.85	33.09	N/A	N/A	N/A	N/A

^a^Day: day of sample collection after the owner test positive for SARS-CoV-2 (Days 1, 3, 5, and 7). ^b^E, RdRp: specific targets for SARS-CoV-2 detection by real-time RT–PCR (E and RdRp genes).  ^*∗*^Virus characterized in this study.

**Table 4:** Genetic mutation of spike gene of SARS-CoV-2 Omicron variant (BA.2) from dog and owner and reference viruses.

Virus	Variant	Accession #	Country	Host	Date	T19R/I	24−27del	A67V	69−70del	T95I	G142D	143−145del	

Wuhan-Hu-1	Wild type	NC_045512.2	China	Human	Dec-19	T	LPPA	A	HV	T	G	no del	
Delta	Delta (B.1.617.2)			Human	Dec-20	R	LPPA	A	HV	T/I	G/D	no del	
CU27791	Delta (B.1.617.2.85)	EPI_ISL_5315539	Thailand	Dog	Sep-21	R	LPPA	A	HV	I	G	no del	
CU27516	Delta (B.1.617.2.30)	EPI_ISL_5320246	Thailand	Cat	Jul-21	R	LPPA	A	HV	T	G	no del	
Omicron BA.1	Omicron (B.1.1.529 + BA.1)			Human	Nov-21	T	LPPA	V	del	I	D	del	
Omicron BA.2	Omicron (B.1.1.529 + BA.2)			Human	Dec-21	I	S	A	HV	T	D	no del	
Omicron BA.4	Omicron (B.1.1.529 + BA.4)			Human	Jan-22	I	S	A	del	T	D	no del	
Omicron BA.5	Omicron (B.1.1.529 + BA.5)			Human	Jan-22	I	S	A	del	T	D	no del	
SUAT_19	Omicron (B.1.1.529 + BA.1.17)	EPI_ISL_11580576	Spain	Cat	Jan-22	T	LPPA	V	del	I	D	del	
TX-014776-001	Omicron (B.1.1.529 + BA.2.3.4)	EPI_ISL_13101428	USA	Dog	Apr-22	I	S	A	HV	T	D	no del	
^*∗*^CU28424	Omicron (B.1.1.529 + BA.2)		Thailand	Dog	Mar-22	I	S	A	HV	T	D	no del	
^*∗*^CUh10001	Omicron (B.1.1.529 + BA.2)		Thailand	Human	Mar-22	I	S	A	HV	T	D	no del	

Virus	Variant	NL211I	V213G	214 ins	G339D	S371L/F	S373P	S375F	T376A	D405N	R408S	K417N	

Wuhan-Hu-1	Wild type	NL	V	no ins	G	S	S	S	T	D	R	K	
Delta	Delta (B.1.617.2)	NL	V	no ins	G	S	S	S	T	D	R	K/N	
CU27791	Delta (B.1.617.2.85)	NL	V	no ins	G	S	S	S	T	D	R	K	
CU27516	Delta (B.1.617.2.30)	NL	V	no ins	G	S	S	S	T	D	R	K	
Omicron BA.1	Omicron (B.1.1.529 + BA.1)	I	V	EPE	D	L	P	F	T	D	R	N	
Omicron BA.2	Omicron (B.1.1.529 + BA.2)	NL	G	no ins	D	F	P	F	A	N	S	N	
Omicron BA.4	Omicron (B.1.1.529 + BA.4)	NL	G	no ins	D	F	P	F	A	N	S	N	
Omicron BA.5	Omicron (B.1.1.529 + BA.5)	NL	G	no ins	D	F	P	F	A	N	S	N	
SUAT_19	Omicron (B.1.1.529+BA.1.17)	I	V	EPE	D	L	P	F	T	D	R	N	
TX-014776-001	Omicron (B.1.1.529 + BA.2.3.4)	NL	G	no ins	N/A	N/A	N/A	N/A	N/A	N/A	N/A	N/A	
^*∗*^CU28424	Omicron (B.1.1.529 + BA.2)	NL	G	no ins	D	F	P	F	A	N	S	N	
^*∗*^CUh10001	Omicron (B.1.1.529 + BA.2)	NL	G	no ins	D	F	P	F	A	N	S	N	

Virus	Variant	N440K	G446S	L452R	S477N	T478K	E484A	F486V	Q493R	G496S	Q498R	N501Y	Y505H

Wuhan-Hu-1	Wild type	N	G	L	S	T	E	F	Q	G	Q	N	Y
Delta	Delta (B.1.617.2)	N	G	R	S	K	E	F	Q	G	Q	N	Y
CU27791	Delta (B.1.617.2.85)	N	G	R	S	K	E	F	Q	G	Q	N	Y
CU27516	Delta (B.1.617.2.30)	N	G	R	S	K	E	F	Q	G	Q	N	Y
Omicron BA.1	Omicron (B.1.1.529 + BA.1)	K	S	L	N	K	A	F	R	S	R	Y	H
Omicron BA.2	Omicron (B.1.1.529 + BA.2)	K	G	L	N	K	A	F	R	G	R	Y	H
Omicron BA.4	Omicron (B.1.1.529 + BA.4)	K	G	R	N	K	A	V	Q	G	R	Y	H
Omicron BA.5	Omicron (B.1.1.529 + BA.5)	K	G	R	N	K	A	V	Q	G	R	Y	H
SUAT_19	Omicron (B.1.1.529 + BA.1.17)	K	S	L	N	K	A	F	R	S	R	Y	H
TX-014776-001	Omicron (B.1.1.529 + BA.2.3.4)	N/A	N/A	N/A	N/A	N/A	A	F	R	G	R	Y	H
^*∗*^CU28424	Omicron (B.1.1.529 + BA.2)	N	G	L	N	K	A	F	R	G	R	Y	H
^*∗*^CUh10001	Omicron (B.1.1.529+BA.2)	N	G	L	N	K	A	F	R	G	R	Y	H

Virus	Variant	T547K	D614G	H655Y	N679K	P681H	N764K	D796Y	N856K	Q954H	N969K	L981F	

Wuhan-Hu-1	Wild type	T	D	H	N	P	N	D	N	Q	N	L	
Delta	Delta (B.1.617.2)	T	G	H	N	R	N	D	N	Q	N	L	
CU27791	Delta (B.1.617.2.85)	T	G	H	N	R	N	D	N	Q	N	L	
CU27516	Delta (B.1.617.2.30)	T	G	H	N	R	N	D	N	Q	N	L	
Omicron BA.1	Omicron (B.1.1.529 + BA.1)	K	G	Y	K	H	K	Y	K	H	K	F	
Omicron BA.2	Omicron (B.1.1.529 + BA.2)	T	G	Y	K	H	K	Y	N	H	K	L	
Omicron BA.4	Omicron (B.1.1.529 + BA.4)	T	G	Y	K	H	K	Y	N	H	K	L	
Omicron BA.5	Omicron (B.1.1.529+ BA.5)	T	G	Y	K	H	K	Y	N	H	K	L	
SUAT_19	Omicron (B.1.1.529 + BA.1.17)	K	G	Y	K	H	K	Y	K	H	K	F	
TX-014776-001	Omicron (B.1.1.529 + BA.2.3.4)	T	G	N/A	N/A	N/A	K	Y	N	H	K	L	
^*∗*^CU28424	Omicron (B.1.1.529 + BA.2)	T	G	Y	K	H	K	Y	N	H	K	L	
^*∗*^CUh10001	Omicron (B.1.1.529 + BA.2)	T	G	Y	K	H	K	Y	N	H	K	L	

^*∗*^Viruses characterized in this study.

**Table 5:** Serological test for SARS-CoV-2 antibodies by sVNT and pVNT in COVID-19 positive dog and owner.

ID	Species	Age	Sample	Serological test									
				cPass (% inhibition)					pVNT (titer)				
				Day 3^a^	Day 7	Day 14	Day 22	Day 31	Day 3	Day 7	Day 14	Day 22	Day 31
CU28424	Dog	1.5 Y		−(1.2%)	+ (94.8%)	−(16.1%)	−(1.4%)	−(9.9%)	−(10)	+ (20)	−(<10)	−(10)	−(10)

CUh10001	Owner	35 Y		N/A	N/A	N/A	N/A	N/A	N/A	N/A	N/A	N/A	N/A

^a^Day: day of sample collection after the owner test positive for SARS-CoV-2 (Days 3, 7, 14, 22, and 31).

## Data Availability

The authors declare that the data supporting the findings of this study are available from the first author upon request. The nucleotide sequence data have been deposited at GenBank with accession numbers: OP862443 and OP862444 and GISAID with accession numbers: EPI_ISL_15833374 and EPI_ISL_15833476.

## Supplementary Materials

Supplementary Materials Table S1: detail of SARS-CoV-2 survey in domestic dogs and cats during the COVID-19 outbreak from December 2021 to August 2022, Thailand. Table S2: genetic mutations of the whole genome of SARS-CoV-2 Omicron variant (BA.2) from dog and owner and reference viruses.
