# Supplementary material for: Serological Evidence of SARS-CoV-2 Exposure in Domestic Dogs and Cats, Thailand: Detection of SARS-CoV-2 Omicron Variant in Dogs Living in COVID-19-Positive Households
Source: Transbound Emerg Dis. 2024 Feb 20;2024:9938523. doi: 10.1155/2024/9938523 (PMC12016992; doi:10.1155/2024/9938523)
Supplement: Supplementary Materials — Table S1: detail of SARS-CoV-2 survey in domestic dogs and cats during the COVID-19 outbreak from December 2021 to August 2022, Thailand. Table S2: genetic mutations of the whole genome of SARS-CoV-2 Omicron variant (BA.2) from dog and owner and reference viruses. [file 9938523.f1.docx]

**Supplement Materials**

**Serological evidence of SARS-CoV-2 exposure in domestic dogs and cats, Thailand:**

**detection of SARS-CoV-2 Omicron variant in dogs living**

**in COVID-19-positive households**

Supassama Chaiyawong^1,2^, Kamonpan Charoenkul^1,2^, Waleemas Jairak^1,2^,

Kitikhun Udom^1,2^, Ekkapat Chamsai^1,2^, Navapon Techakriengkrai ^1,3^,

Kamol Suwannakarn^4^, Alongkorn Amonsin^1,2*^

^1^Department of Veterinary Public Health, Faculty of Veterinary Science, Chulalongkorn University, Bangkok, 10330, Thailand

^2^Emerging and Re-emerging Infectious Diseases in Animals, Center of Excellence,

Faculty of Veterinary Science, Chulalongkorn University, Bangkok, 10330, Thailand

^3^Department of Veterinary Microbiology, Faculty of Veterinary Science, Chulalongkorn University, Bangkok, Thailand

^4^Department of Microbiology, Faculty of Medicine Siriraj Hospital, Mahidol University, Bangkok, Thailand

**Supplement tables**

**Supplement Table 1.** Detail of SARS-CoV-2 survey in domestic dogs and cats during the COVID-19 outbreak from December 2021 to August 2022, Thailand.

**Supplement Table 2.** Genetic mutations of the whole genome of SARS-CoV-2 Omicron variant (BA.2) from dog and owner and reference viruses

**Supplement Table 1.** Detail of SARS-CoV-2 survey in domestic dogs and cats during the COVID-19 outbreak from December 2021 to August 2022, Thailand.

| Month | COVID-19 Households | | | Unknown status Households | |
| --- | --- | --- | --- | --- | --- |
|  | #Household | Dog | Cat | Dog | Cat |
|  |  | #positive/#tested | #positive/#tested | #positive/#tested | #positive/#tested |
| Dec 2021 | 3 | 0/4 | 0/2 | 0/2 | 0/1 |
| Jan 2022 | 1 | 0/1 | 0/0 | 0/2 | 0/4 |
| Feb 2022 | 1 | 0/1 | 0/0 | 0/15 | 0/13 |
| Mar 2022 | 10 | 1/16 | 0/4 | 0/32 | 0/17 |
| Apr 2022 | 6 | 0/3 | 0/10 | 0/3 | 0/5 |
| May 2022 | 3 | 0/61 | 0/7 | 0/41 | 0/12 |
| Jun 2022 | 8 | 0/1 | 0/14 | 0/65 | 0/13 |
| July 2022 | 11 | 0/8 | 0/9 | 0/43 | 0/15 |
| Aug 2022 | 6 | 0/1 | 0/14 | 0/18 | 0/15 |
| **Total** | **49** | **1/96** | **0/60** | **0/221** | **0/95** |

**Supplement Table 2.** Genetic mutations of the whole genome of SARS-CoV-2 Omicron variant (BA.2) from dog and owner and reference viruses

| **Virus** | **Variant** | **Accession #** | **Country** | **Host** | **Date** | **ORF1ab** |  |  |  |  |  |  |
| --- | --- | --- | --- | --- | --- | --- | --- | --- | --- | --- | --- | --- |
|  |  |  |  |  |  | S135R | 141-143del | T842I | K856R | G1307S | SL2083I | A2710T |
| Wuhan-Hu-1 | Wild‐type | NC_045512.2 | China | Human | Dec-19 | S | no del | T | K | G | SL | A |
| Delta | Delta (B.1.617.2) |  |  | Human | Dec-20 | S | no del | T | K | G | SL | A |
| CU27791 | Delta (B.1.617.2.85) | EPI_ISL_5315539 | Thailand | Dog | Sep-21 | S | no del | T | K | G | SL | A |
| CU27516 | Delta (B.1.617.2.30) | EPI_ISL_5320246 | Thailand | Cat | Jul-21 | S | no del | T | K | G | SL | A |
| Omicron BA.1 | Omicron (B.1.1.529+BA.1) |  |  | Human | Nov-21 | S | no del | T | R | G | I | T |
| Omicron BA.2 | Omicron (B.1.1.529+BA.2) |  |  | Human | Dec-21 | R | no del | I | K | S | SL | A |
| Omicron BA.4 | Omicron (B.1.1.529+BA.4) |  |  | Human | Jan-22 | R | del | I | K | S | SL | A |
| Omicron BA.5 | Omicron (B.1.1.529+BA.5) |  |  | Human | Jan-22 | R | no del | I | K | S | SL | A |
| SUAT_19 | Omicron (B.1.1.529+BA.1.17) | EPI_ISL_11580576 | Spain | Cat | Jan-22 | S | no del | T | R | G | I | T |
| TX-014776-001 | Omicron (B.1.1.529+BA.2.3.4) | EPI_ISL_13101428 | USA | Dog | Apr-22 | R | no del | I | K | S | SL | A |
| *CU28424 | Omicron (B.1.1.529+BA.2) |  | Thailand | Dog | Mar-22 | R | no del | N/A | N/A | N/A | SL | A |
| *CUh10001 | Omicron (B.1.1.529+BA.2) |  | Thailand | Human | Mar-22 | R | no del | I | K | S | SL | A |
|  |  |  |  |  |  |  |  |  |  |  |  |  |
| **Virus** | **Variant** | **ORF1ab** |  |  |  |  |  |  |  |  |  |  |
|  |  | L3027F | T3090I | L3201F | T3255I | P3395H | 3675-3677del | I3758V | P4715L | R5716C | I5967V | T6564I |
| Wuhan-Hu-1 | Wild‐type | L | T | L | T | P | no del | I | P | R | I | T |
| Delta | Delta (B.1.617.2) | L | T | L | T | P | no del | I | P | R | I | T |
| CU27791 | Delta (B.1.617.2.85) | L | T | L | I | P | no del | I | L | R | I | T |
| CU27516 | Delta (B.1.617.2.30) | L | T | L | T | P | no del | I | L | R | I | T |
| Omicron BA.1 | Omicron (B.1.1.529+BA.1) | L | T | L | I | H | del | V | L | R | V | T |
| Omicron BA.2 | Omicron (B.1.1.529+BA.2) | F | I | F | I | H | del | I | L | C | V | I |
| Omicron BA.4 | Omicron (B.1.1.529+BA.4) | F | I | L | I | H | del | I | L | C | V | I |
| Omicron BA.5 | Omicron (B.1.1.529+BA.5) | F | I | L | I | H | del | I | L | C | V | I |
| SUAT_19 | Omicron (B.1.1.529+BA.1.17) | L | T | L | I | H | del | V | L | R | V | T |
| TX-014776-001 | Omicron (B.1.1.529+BA.2.3.4) | F | I | F | I | H | del | I | L | C | V | I |
| *CU28424 | Omicron (B.1.1.529+BA.2) | N/A | I | F | I | H | del | I | L | C | V | I |
| *CUh10001 | Omicron (B.1.1.529+BA.2) | F | I | F | I | H | del | I | L | C | V | I |
|  |  |  |  |  |  |  |  |  |  |  |  |  |
| Virus | Variant | **N** |  |  |  |  |  |  | **ORF3a** | **E** | **M** |  |
|  |  | P13L | 31-33del | P151S | R203K | G204R | 366del | S413R | T223I | T9I | D3G/N | Q19E |
| Wuhan-Hu-1 | Wild‐type | P | no del | P | R | G | no del | S | T | T | D | Q |
| Delta | Delta (B.1.617.2) | P | no del | P | M | G | no del | S | T | T | D | Q |
| CU27791 | Delta (B.1.617.2.85) | P | no del | P | M | G | no del | S | T | T | D | Q |
| CU27516 | Delta (B.1.617.2.30) | P | no del | P | M | G | no del | S | T | T | D | Q |
| Omicron BA.1 | Omicron (B.1.1.529+BA.1) | L | del | P | K | R | no del | S | T | I | G | E |
| Omicron BA.2 | Omicron (B.1.1.529+BA.2) | L | del | P | K | R | no del | R | I | I | D | E |
| Omicron BA.4 | Omicron (B.1.1.529+BA.4) | L | del | S | K | R | no del | R | I | I | D | E |
| Omicron BA.5 | Omicron (B.1.1.529+BA.5) | L | del | P | K | R | no del | R | I | I | N | E |
| SUAT_19 | Omicron (B.1.1.529+BA.1.17) | L | del | P | K | R | no del | S | T | I | G | E |
| TX-014776-001 | Omicron (B.1.1.529+BA.2.3.4) | L | del | P | K | R | no del | R | I | I | D | E |
| *CU28424 | Omicron (B.1.1.529+BA.2) | L | del | P | K | R | del | R | I | I | D | E |
| *CUh10001 | Omicron (B.1.1.529+BA.2) | L | del | P | K | R | del | R | I | I | D | E |
|  |  |  |  |  |  |  |  |  |  |  |  |  |
| Virus | Variant | **M** | **ORF6** | **ORF7b** | **ORF9b** |  |  |  |  |  |  |  |
|  |  | A63T | D61L | L11F | P10S | 27-29del |  |  |  |  |  |  |
| Wuhan-Hu-1 | Wild‐type | A | D | L | P | no del |  |  |  |  |  |  |
| Delta | Delta (B.1.617.2) | A | D | L | P | no del |  |  |  |  |  |  |
| CU27791 | Delta (B.1.617.2.85) | A | D | L | P | no del |  |  |  |  |  |  |
| CU27516 | Delta (B.1.617.2.30) | A | D | L | P | no del |  |  |  |  |  |  |
| Omicron BA.1 | Omicron (B.1.1.529+BA.1) | T | D | L | S | del |  |  |  |  |  |  |
| Omicron BA.2 | Omicron (B.1.1.529+BA.2) | T | L | L | S | del |  |  |  |  |  |  |
| Omicron BA.4 | Omicron (B.1.1.529+BA.4) | T | L | F | S | del |  |  |  |  |  |  |
| Omicron BA.5 | Omicron (B.1.1.529+BA.5) | T | D | L | S | del |  |  |  |  |  |  |
| SUAT_19 | Omicron (B.1.1.529+BA.1.17) | T | D | L | S | del |  |  |  |  |  |  |
| TX-014776-001 | Omicron (B.1.1.529+BA.2.3.4) | T | L | L | S | del |  |  |  |  |  |  |
| *CU28424 | Omicron (B.1.1.529+BA.2) | T | L | L | S | del |  |  |  |  |  |  |
| *CUh10001 | Omicron (B.1.1.529+BA.2) | T | L | L | S | del |  |  |  |  |  |  |
